# Model-Based Localization and Tracking Using Bluetooth Low-Energy Beacons

**DOI:** 10.3390/s17112484

**Published:** 2017-10-29

**Authors:** F. Serhan Daniş, Ali Taylan Cemgil

**Affiliations:** 1 Department of Computer Engineering, Boğaziçi University, Istanbul 34342, Turkey; taylan.cemgil@boun.edu.tr; 2 Department of Computer Engineering, Galatasaray University, Istanbul 34349, Turkey

**Keywords:** Bluetooth low-energy localization, hidden Markov model, BLE tracking, observation probability estimation, Wasserstein distance, Wasserstein interpolation, affine Wasserstein combination, sequential Monte Carlo

## Abstract

We introduce a high precision localization and tracking method that makes use of cheap Bluetooth low-energy (BLE) beacons only. We track the position of a moving sensor by integrating highly unreliable and noisy BLE observations streaming from multiple locations. A novel aspect of our approach is the development of an observation model, specifically tailored for received signal strength indicator (RSSI) fingerprints: a combination based on the optimal transport model of Wasserstein distance. The tracking results of the entire system are compared with alternative baseline estimation methods, such as nearest neighboring fingerprints and an artificial neural network. Our results show that highly accurate estimation from noisy Bluetooth data is practically feasible with an observation model based on Wasserstein distance interpolation combined with the sequential Monte Carlo (SMC) method for tracking.

## 1. Introduction

In this work we address the problem of positioning and tracking using beacons that transmit data with Bluetooth low-energy (BLE) messages. In the most general setup, we distribute beacons whose exact positions are not necessarily known in a closed indoor area, and we need to estimate and track the position of a mobile sensor using BLE messages emitted by the beacons. As the underlying technology uses short wavelength radio signals, data transfer is affected by a high number of factors, such as scattering and reflections, all of which can not be exactly known. We attempt to model and solve this issue using probabilistic methods.

BLE is a distinctive feature that was added to the Bluetooth technology standard at v4.0. It has been introduced to facilitate short-range communication, which requires large amounts of data transfer. However, this new feature in Bluetooth technology made it a good candidate as part of positioning systems as it provides an efficient way of transferring messages with low energy, with the help battery-powered mobile beacons to emit messages for durations in the order of years [1]. Moreover, BLE technology provides the variables that can be used to predict the position information. One of those variables is the received signal strength indicator (RSSI), which may give an estimate of the flight distance of the signal from the beacon to the receiver [2]. Neburka et al. [3], after extensively analyzing the performance of BLE technology in indoor environments, show that it is a promising technique for indoor positioning, even though the RSSI values are inaccurate and highly depend on the BLE module used. Its new abilities, like durability, mobility, and high reaction time have led to the Bluetooth BLE technology replacing Wi-Fi for positioning purposes.

An accurate, lightweight, and easily deployable tracking system for indoor environments has many practical applications. For example, in marketing and retail, a reliable indoor positioning and tracking system (IPS) deployed in a closed area such as a mall or a shopping center, is used for targeted advertising, campaign management and customer behavior monitoring [4,5,6]. In healthcare, elderly people or patients with dementia can be tracked in indoor environments without needing any other caretaking personnel or complex sensors [7]. In a factory or production environment, assets or hardware can be continuously logged for security reasons or inventory analysis [8]. In mobile robotics, localization is the basis for trajectory planning and smooth and secure navigation. Researchers attempt to solve this problem with dead-reckoning using inertial sensors accompanied by visual/range-related environmental sensors like cameras, range scanners, and sonars, or use a combination of these methods [9,10]. BLE fits into this domain as another practical, simple, and economical sensory system.

There are mainly two basic techniques used in IPS: *trilateration* and *fingerprinting*: In *trilateration*, estimated distances are used to calculate the most probable coordinates using the geometry of triangles [11]. With BLE, the distances to the beacons are estimated via the RSSI readings which are expected to map to the actual distances according to the *radio frequency* (RF) propagation model [12]. Chen et al. [13,14] combine the pedesterian dead reckoning (PDR) with a weighted path loss (WPL) algorithm that bases on the log-distance path loss model between a router and a client, under an extended Kalman filter. While the technique is based on a fast training phase, problems arise due to the RSSI-to-distance mapping model, which is regarded unstable due to the unreliable distance estimation in BLE [15,16]. On the other hand, *fingerprinting* relies on a prior scene analysis (or radio map construction [17]), in which measurements are collected from the environment and calibrated into specific features, called fingerprints, which describe a part of the environment [18]. The system estimates the location of a sensor by comparing the online measurements with the fingerprints, and the most similar fingerprint location is regarded as an estimate for the expected position. To make the technique robust and precise, the fingerprint locations must be as dense as possible. Thus, there arises a trade-off between the installation overhead versus the precision. Besides, the overhead may also include the recalibration after the system is installed [19,20]. Previous work on IPS shows that fingerprinting-based techniques outperform the trilateration technique with respect to reliability and precision [16,21,22].

Different algorithms are employed over the above mentioned IPS techniques. The authors of [23] study three typical algorithms in IPS domain: the k-nearest neighbors (kNN), neural networks and support vector machines (SVM). They show that kNN methods yield better results for localization than the other two. The authors of [24] use fingerprint technique and evaluate BLE localization performances based on extensive indoor measurements, mapped by propagation modes. The authors of [25] combine the propagation model (PM) with the extended Kalman filter (EKF), and reach an error rate of 2.56 m, improving the localization accuracy with sparse beacon deployment. The authors of [26] compare the fingerprinting technique via Wi-Fi with the current fingerprinting via BLE. They use a Bayesian estimator as the main method for positioning with a grid size of 1 m, and achieve a tracking accuracy of 2.6 m using a dense beacon distribution and 4.8 m using a sparse distribution, which, as they claim, is a significant improvement over Wi-Fi even using sparse beacon deployment [27]. As another technique, Chen et al. [28] study the Bayesian fusion methods using Bluetooth fingerprints and achieve a horizontal positioning accuracy of 4.7 m on average with respect to Bayesian static estimation and Kalman filter.

**Figure 1:**
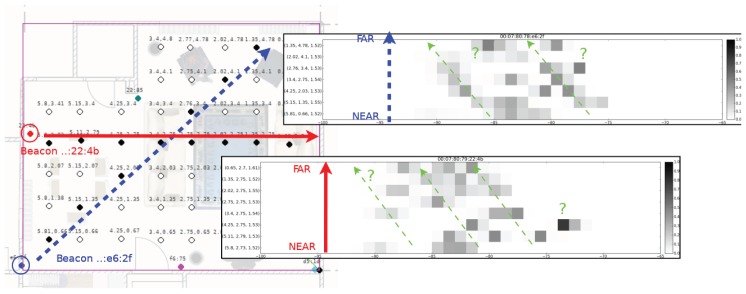
Change in the histograms with respect to distance.

Related literature guides us to use the fingerprinting technique. Nevertheless, we attempt to see if the trilateration technique may be applied with the beacon set in hand. As previous research [16] suggests, we ask if we can fit a logarithm-based attenuation model to the RSSI data so that we can map the data to a distance value easily, but without even attempting to perform a fitting, we can see the inconsistency in the histograms changing with distance. Figure 1 shows the points that move away from two different beacons and their corresponding histograms of the RSSI data on each position. While the expected behavior of the modes would be to move from lower values to higher values as the point gets farther away from the beacon, we cannot visualize this behavior consistently. The direction of the blue arrow is selected to be aligned with almost linearly positioned fingerprint locations, so that we expect a consistent attenuation of strength values of the corresponding beacon data. The histograms of the blue beacon on Figure 1 do not display the expected behavior. Instead, we see more than one peaked histograms as we get farther away. The same inconsistency can be visualized with the similar setting for the red beacon.

As we cannot map the data to some distance metric, the trilateration method does not seem to be an option in our case. Even when the beacon is in the *line-of-sight* (LoS) of the sensor, the data values show interesting fluctuations. Moreover, a single reading, which may be a reflection, would lead to incorrect distance mappings, but with fingerprinting the same reading can be explained by another mode of a histogram.

**Figure 2:**
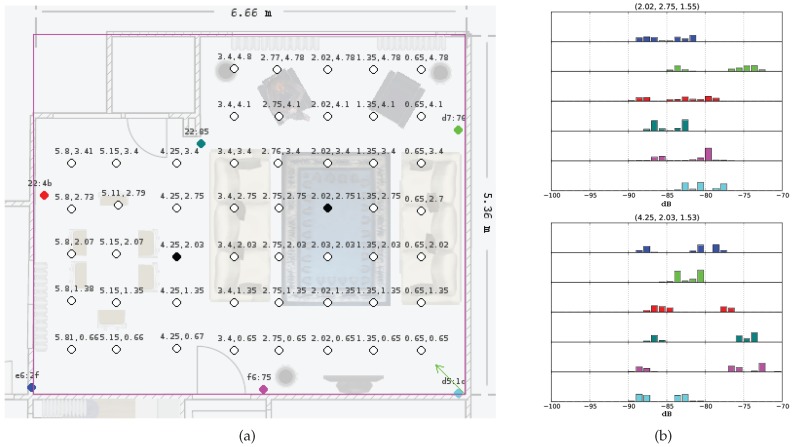
Sample fingerprints and their positions. (**a**) A dense fingerprinting example in an indoor environment. Hollow, black and colored dots show measurement positions, sample fingerprint positions (**b**) and beacon positions, respectively. (**b**) Sample fingerprints (histograms) on different locations for different beacons. Color codes are the same with the beacon dot colors in (**a**).

Accordingly in this work, we rely on the fingerprinting technique and we compute the histograms of RSSI readings on certain positions as the fingerprints (see Figure 2a), but we cannot use the conventional tracking algorithms like the Kalman filter or its variations as the fingerprint histograms do not display any Gaussian-like structure, possibly because of the signal reflections from various objects and surfaces (see Figure 2b). Moreover, we could have discarded the data of the lower strengths by using sliding windows to arrive at Gaussian-like histograms, but we have the tendency to keep the multimodality in the histograms, believing that the multimodality has the location information within.

However, for fine localization, we require many fingerprints, the collection of which costs time and thus is impractical. Vector interpolation techniques are considered to estimate the histograms associated with arbitrary map positions. Such a technique is applied in the works of [29,30] with Wi-Fi strength indicators. They approximate the likelihood of an observation via Nadaraya–Watson kernel regression, which is a computationally complex model, but they achieve accuracies under one meter with Wi-Fi.

We introduce a histogram interpolation technique, affine Wasserstein histogram interpolation, into the IPS domain to approximate a radio map using lower number of fingerprints, which is obviously less time consuming and more practical. With an intelligent interpolation, sparsely distributed fingerprints are used to estimate the densities at any location. Our radio map model differs from the state-of-the-art by its tendency to define the interpolated histograms by a combination of the surrounding histograms, which makes the method totally data driven, and thus the output is expected to keep the original modes of the histograms (because of scattering and reflections).

The Wasserstein distance is originally a distance metric in optimal transport, defining the distance between two distributions [31]. While optimally transporting measures between distributions, a transport map is calculated and the measures are gradually moved according to this map [32,33], which corresponds to an interpolation between two distributions. The authors of [34] use a similar regression intuition in computer graphics under the name `Wasserstein Barycenter coordinates’. One of their applications is recolorizing the images with a transition between the original and the objective color histograms. Likewise, the authors of [35] introduce a class of algorithms for tractable optimization problems to solve optimal transportation over geometric domains and apply the idea for shape interpolation, Bidirectional Reflectance Distribution Function (BRDF) design, color histogram manipulation, skeleton layout, and soft maps.

With a radio map approximation model in hand, we require a tracking method to estimate the positions. For this purpose, the authors of [36] employ the article filter for indoor localization fusing Wi-Fi- received signal strengths with accelerometer, gyroscope, map, and barometer data. They show that map information provides a significant improvement on the positioning accuracy. The authors of [37] use the particle filter over the iBeacon data. They achieve an error rate as low as 0.27 m using the trilateration technique.

For the localization purposes, the SMC method can be employed if we can model the motion and have a location related evaluation [38]. As we have both, we build an SMC method to tackle the problem of indoor positioning and tracking. However, the main contribution of this work is the application of Wasserstein interpolation to estimate the observation densities that map the positions to the beacon RSSI densities. As side contributions, we also add the other regression methods, like the k-nearest neighbor convex combination and neural networks to estimate the observation densities on arbitrary map coordinates. The estimated observation densities are then used to in the update step of the SMC algorithm.

In Section 2, we give the details on the tracking algorithms and the accessories used in these algorithms. Section 3 describes the test setup and the experiments. We give the experimental results in Section 4 and finalize our report with a summary and a plan of the future of the research in Section 5.

## 2. Methodology

We are dealing with an indoor positioning and tracking problem. This section shows the model of this problem, a hidden Markov model (HMM), followed by a simple transition density model. We focus primarily on the estimation of the observation densities on arbitrary positions, so that the likelihood of a single position can be computed. While we contribute to the domain with the application of Wasserstein interpolation to estimate observation density, many other regression or interpolation methods are also discussed. With transition and observation densities in hand, we show how to construct an SMC filter for BLE localization and tracking at the end of the section.

In the most general case, bold uppercase letters (T,H) denote matrices, and bold lowercase letters (x,y,α) are the vectors. Concordantly, h will always symbolize the histogram vectors. An element of a vector will be denoted with the unbold version of the original vector and with an index in the subscript. Index numbering is classically in lower case. Particularly, *t* always denotes the time points. The superscript on the histogram ($h^{P}$) symbolizes the position it is generated on. If the histogram is estimated by a specific method, it will be marked with a tilde (∼), the method will be written in the subscript, and possible hyperparameters will be written as parameters to the method function (${\tilde{h}}_{\mathrm{NF}}^{P}\left(k\right)$). The positions are of two types: *P*, the arbitrary ones, and *F*, the fingerprint positions. Fingerprint positions will be indexed in the subscript as they belong to a finite set. p(x|y,Θ) denote the conditional probability distributions, and Θ are the problem specific hyperparameters that, in this work, depend on the estimation algorithms.

### 2.1. Tracking Problem

**Figure 3:**
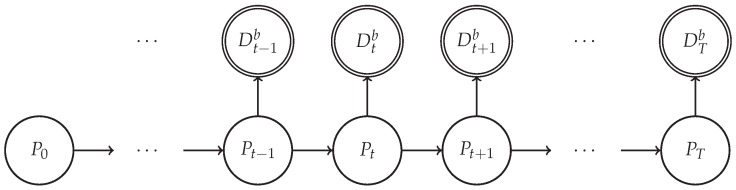
Graphical model for tracking.

We form a hidden Markov model (HMM) for the tracking problem. Let A be a subset of $R^{2}$ and P=(x,y) be a position in this set A, forming the latent variables. We observe the RSSI values belonging to a discrete set of {−120,…,−60}, the size of which is adjusted according to the maximal values of the captured RSSI values. We denote each data instance $D_{t}^{b}$, with *t* being the time stamp of such an instance and *b* being the index of the beacon that transmits data. According to our model $D_{t}^{b}$ depends on the position $P_{t}$ and the beacon $b_{t}$. The generative model follows and the graphical model is given in Figure 3:

(1) $$\begin{array}{c} \begin{array}{cc} D_{t}^{b} & ∼p(D_{t}^{b}|P_{t},\Theta )\\ P_{0} & ∼p(P_{0})\\ P_{t} & ∼p(P_{t}|P_{t-1}) \end{array} \end{array}$$

For the transition density, $p(P_{t}|P_{t-1})$, we use the motion model given in Section 2.2. In Section 2.3, we construct the estimators for the observation density via multiple methods that use the fingerprints. With the estimated observation densities we compute the likelihood of a new observation sample, $p(D_{t}^{b}|P_{t},\Theta )$. The two models are then combined in a tracking filter described in Section 2.4.

### 2.2. Transition Model (Diffusion Motion Model)

**Figure 4:**
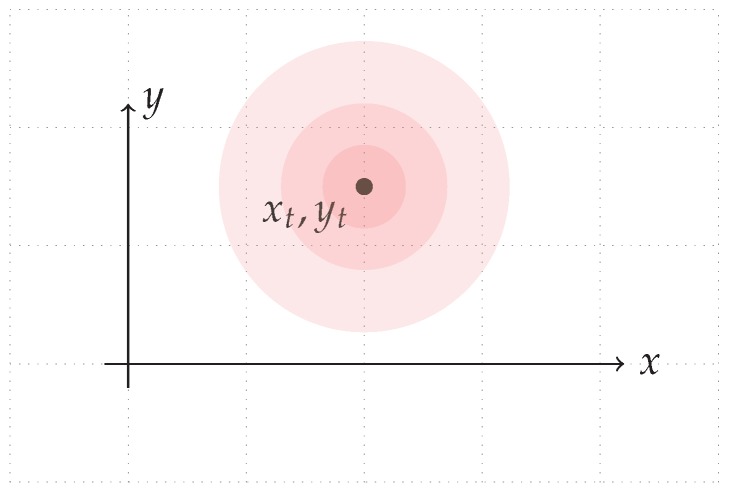
Distribution of the position of a mobile agent at time *t*.

We restrict our agent to reside on a plane, in which the position is composed of two Cartesian coordinates, $x_{t}=\left(x_{t},y_{t}\right)$ (see Figure 4).

We assume that the robot is not fed by any internal sensory data that enables dead reckoning. The diffusion motion model relies on the assumption that the robot will be in a close position to a previous one, which is thus modeled as a Gaussian distribution with the previous position as the mean value. The covariance of the distribution determines how far the robot can move in a time unit [10].

The next state, $x_{t}$, can be modeled by:

(2) $${\left(\begin{array}{c} x\\ y \end{array}\right)}_{t}={\left(\begin{array}{c} x\\ y \end{array}\right)}_{t-1}+\left(\begin{array}{c} \varepsilon_{x}\\ \varepsilon_{y} \end{array}\right)$$

where $\varepsilon_{i}=N\left(0,R\right)$. We assume that distributions on individual dimensions are independent and their variances are equal (*R*).

### 2.3. Observation Models

In this section, we list our contributions for the observation models to find an estimate on the likelihood of a measurement given a position of the map.

#### 2.3.1. Nearest Fingerprint (*NF*)

The most naïve method to estimate the likelihood density on a position *P* is to use directly the information on the nearest fingerprint position, corresponding to the *k*-nearest neighbor method with k=1. For a given position, *P*, we find the nearest fingerprint position to *P* with respect to the Euclidean distance. With $F_{i}\in F$ and i∈[1..N]:

(3) $$\begin{array}{c} i^{*}=\arg\min_{i}∥P-F_{i}∥ \end{array}$$

We use the corresponding histogram on the closest fingerprint location, $h^{F_{i^{*}}}$, as the likelihood:

(4) $$\begin{array}{c} p\left(D_{b}|P,\Theta \right)={\tilde{h}}_{\mathrm{NF}}^{P}=h^{F_{i^{*}}} \end{array}$$

This method assumes that the area in which the position estimation will be handled is densely sampled, so that for every position *P*, there happens to be a close fingerprint position.

#### 2.3.2. k-Nearest Fingerprint Combination (*kNF*)

**Figure 5:**
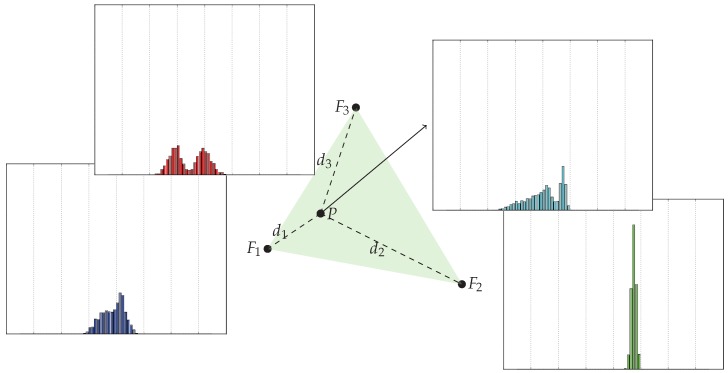
Visualization of a convex combination at the position *P* in the convex hull of some other fingerprint positions.

Alternatively, we can take the convex combination of the surrounding fingerprints. We compute a linear combination of the histograms with respect to their distances to the estimation position which reside in the convex hull of the input histograms (see Figure 5). Furthermore, we generalize the convex combination onto the plane spanned by the fingerprint positions, relaxing the convex hull to an affine hull, making it an affine vector combination.

Let $F_{j}$ denote the fingerprint positions on which the histograms are previously computed, $h^{F_{j}}$, and let the position *P* reside in the affine hull of a subset of fingerprint positions. Then the histogram on *P*, $h^{P}=\left\{h_{i}\right\}$, is defined as:

(5) $$\begin{array}{c} h_{i}=\sum_{j}\lambda_{j}h_{i}^{F_{j}} \end{array}$$

where $\lambda =\{\lambda_{j}\}$ are multipliers that are tuned with respect to the distances between the estimation position *P* and the fingerprint positions $F_{j}$, and necessarily $\sum_{j}\lambda_{j}=1$. In particular, we use the softmax function to boost the effect of the close fingerprint positions: $\lambda_{j}=\frac{\exp(-∥P-F_{j}∥)}{\sum_{k}\exp(-∥P-F_{k}∥)}$. The observation density of a specific position on the map can then be estimated as

(6) $$\begin{array}{c} p\left(D_{b}|P,\Theta \right)={\tilde{h}}_{\mathrm{kNF}}^{P}\left(k\right) \end{array}$$

where *k* is a parameter that decides how many fingerprint positions closest to the estimation position will be used. Note that the method *NF* is the special case of *kNF* with k=1.

#### 2.3.3. The Artificial Neural Network (*ANN*)

**Figure 6:**
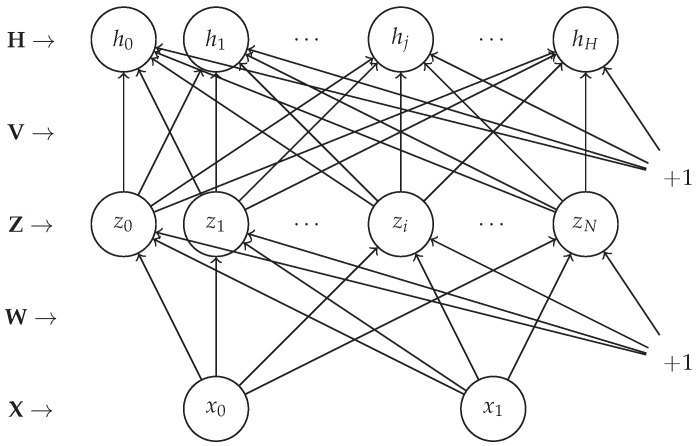
Neural network structure for histogram estimation.

Considering the problem as a regression, the intuition leads to tackle it using the neural network approach. A multilayer neural network takes the map coordinates as the input, and returns the histogram measures at that coordinates as the output. We expect the synaptic links to learn the histograms with respect to the positions (see Figure 6).

Layers are fully connected via nonlinear activation functions (hyperbolic tangent and sigmoid) of the linear perceptron equations. We also add a bias for each node to make the structure learn an offset value if possible. The forward neural equations in matrix notation are given in (7).

(7) $$\begin{array}{cc} Z & =\tanh\left(\left[X\;1\right]W\right)\\ H & =\mathrm{sigm}\left(\left[Z\;1\right]V\right) \end{array}$$

where X is of size N×(D+1), W of (D+1)×M, Z of N×(M+1), V of (M+1)×L, and H of N×L, and the sizes N,D,M and *L* denote the sample size, number of inputs (coordinates), hidden layer size, and number of outputs (histogram indices), respectively.

We employ the Keras Deep Learning Libary [39] for training the neural network model and find the interlayer weights. The algorithm starts with uniformly randomized weights with values in [−0.05,0.05]. The first layer is passed through the hyperbolic tangent and the second layer through the sigmoid function as the relations are considered to be nonlinear. We use the RMSprop, an adaptive gradient descent-based learning rate method as the optimizer [40], and the mean squared error as the loss function.

With the weights in hand after training, we run the network in the forward direction to get a histogram estimation for the position of interpolation, *P*:

(8) $$\begin{array}{c} p\left(D_{b}|P,\Theta \right)={\tilde{h}}_{\mathrm{ANN}}^{P}\left(N_{h}\right) \end{array}$$

where $N_{h}$ is the size of the hidden layer.

#### 2.3.4. Affine Wasserstein Combination (*AWC*)

The Wasserstein distance is originally a distance metric used to compare densities [32,33]. In the process of calculating the cost to transport the measures between densities, we also obtain a transport function that maps the measures of one density to the measures of the other one. We employ the transport function to produce an interpolation by transporting the mesaures gradually and to develop an affine combination between two or more histogram positions.

In a discrete state space, we replace the transport function, T(x) with a transport matrix, $T=\{\tau_{i,j}\}$, which represents the measure to be transported from a one dimensional vector, $x=\{x_{i}\}$, to another one dimensional vector, $y=\{y_{i}\}$. In our case, these vectors are actually normalized histograms, whose bins are identical and each bin index corresponds to an integer between the given extremities.

$$\begin{array}{c} \begin{array}{c} x=\{x_{i}\}\\ y=\{y_{i}\} \end{array}\;\mathrm{where}\;\begin{array}{c} 0\leq x_{i}\leq 1\\ 0\leq y_{i}\leq 1 \end{array}\;\mathrm{and}\;\begin{array}{c} \sum_{i}x_{i}=1\\ \sum_{i}y_{i}=1 \end{array} \end{array}$$

In the discrete case [41], the Wasserstein distance is the minimum cost of transporting one histogram onto another:

$$\begin{array}{cc} W(x,y) & =\min_{W\in W_{x,y}}\sum_{i,j}\tau_{i,j}c\left(i,j\right) \end{array}$$

where $\tau_{i,j}$ is the measure that would be transported from the location *i* to the location *j* and c(i,j) is the cost multiplier that is usually related to the distance between the locations i,j.

If the cost function, *c*, is a linear function, the Wasserstein transport matrix is efficiently computed by scanning the array and keeping track of the transported weights between bins.

##### Wasserstein Interpolation

**Figure 7:**
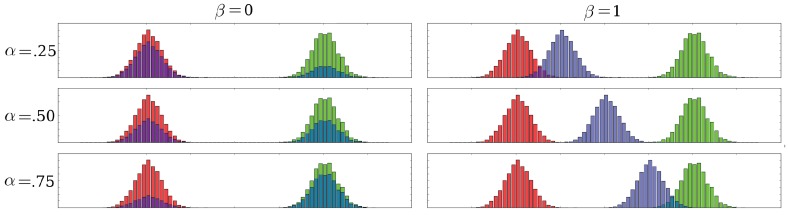
Interpolation ambiguity: We want to move the pile of red histogram onto the green histogram. The figure on the left represents a direct jump of the measures onto the new location, whereas the one on the right shows a gradual movement of the measures towards the new pile location.

We can perform the interpolation operation in multiple ways (Figure 7). In fact, we define two parameters, α and β, both in the interval of [0,1] [33]. The actual interpolation between densities is controlled by the coefficient α, which decides how similar would the interpolation be to the original densities. For the values of α near zero, the interpolation will be similar to the source histogram, whereas for the higher values will make the interpolation resemble the destination histogram. β controls the evolution of the interpolaton between a linear interpolation (β=0) and a displacement interpolation (β=1) [33].

Formally, given two histograms, namely $h^{0}$ and $h^{1}$, we first compute the Wasserstein mapping, $T_{h^{0},h^{1}}$, which is merely a matrix that demonstrates the transport plan of the measures. According to this transport plan, we calculate two intermediate plans, $I_{\alpha ,\beta }^{\prime }=\left\{\iota_{i,j}^{\prime }\right\}$ and $I_{\alpha ,\beta }^{\prime \prime }=\left\{\iota_{i,j}^{\prime \prime }\right\}$, which represent the destination indices. The measures, $\tau_{i,j}$ will be distributed to:

(9) $$\begin{array}{c} \begin{array}{cc} \iota_{i,j}^{\prime } & =i+\lceil \alpha \beta (j-i)\rceil \\ \iota_{i,j}^{\prime \prime } & =j-\lceil (1-\alpha )\beta (j-i)\rceil \end{array} \end{array}$$

where ⌈.⌉ is the ceil operator as the indices should be integers. Indices of the final interpolation are computed as follows:

(10) $$\begin{array}{cc} h_{k} & =\sum_{i}\sum_{j}\left(1-\alpha \right)\tau_{i,j}\delta \left(\iota_{i,j}^{\prime }=k\right)+\alpha \tau_{i,j}\delta \left(\iota_{i,j}^{\prime \prime }=k\right) \end{array}$$

where δ∈{0,1} is the Kronecker delta operator.

Note that a two-position convex combination (*kNF*) of histograms is merely a special case of the Wasserstein combination, in which β is set to 0.

##### Affine Wasserstein Combination

The interpolation of histograms can be generalized to their linear combinations by varying α on the real line, relaxing the limitation of the interval [0,1] to acquire values out of the interval. With the intermediate transport plans (9), we redefine the interpolation operation at (10) as:

(11) $$\begin{array}{c} \begin{array}{cc} h_{k} & =\sum_{i}\sum_{j}\frac{\left|1-\alpha \right|}{\left|1-\alpha |+|\alpha \right|}\tau_{i,j}\delta \left(\iota_{i,j}^{\prime }=k\right)\\ & +\frac{\left|\alpha \right|}{\left|1-\alpha |+|\alpha \right|}\tau_{i,j}\delta \left(\iota_{i,j}^{\prime \prime }=k\right) \end{array} \end{array}$$

**Figure 8:**
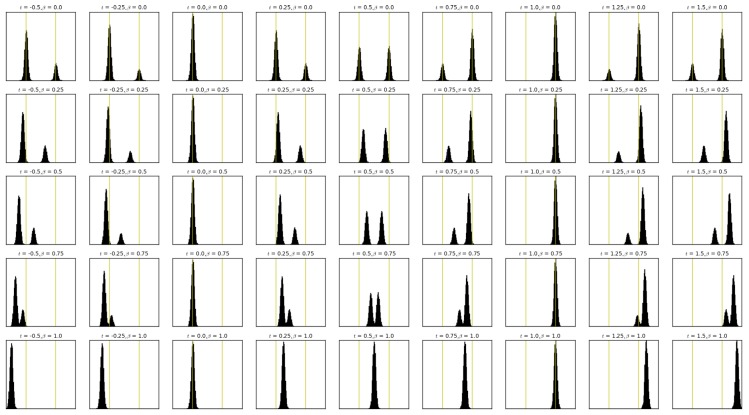
Affine combination of two histograms of Gaussian densities for different α and β values. Yellow vertical lines show the original location of the modes.

The algorithm is given in Algorithm 1 and the combinations for different values of α and β can be seen in Figure 8.

**Algorithm 1** Affine Wasserstein Histogram Combination
1:Initial two histograms $h^{0}=\left\{h_{i}^{0}\right\}$ and $h^{1}=\left\{h_{j}^{1}\right\}$.2:Calculate the discrete Wasserstein mapping $T=\{\tau_{i,j}\}$.3:Initialize the interpolation h with zeros, $h_{k}=0$.4:**for all** i,j **do**5: Calculate new histogram indices of the measure$\;k_{0}=i+\left\lceil \alpha \beta \left(j-i\right)\right\rceil$$\;k_{1}=j-\left\lceil \left(1-\alpha \right)\beta \left(j-i\right)\right\rceil$6: Distribute the weight on these indices$\;h_{k_{0}}=h_{k_{0}}+\frac{\left|1-\alpha \right|}{\left|1-\alpha |+|\alpha \right|}\tau_{i,j}$$\;h_{k_{1}}=h_{k_{1}}+\frac{\left|\alpha \right|}{\left|1-\alpha |+|\alpha \right|}\tau_{i,j}$7:**end for**8:**return**  h


##### Pair Selection Strategy (Loose Alignment)

**Figure 9:**
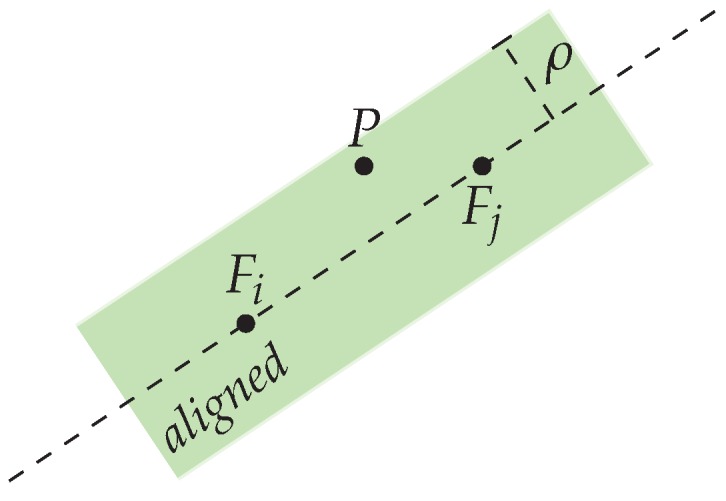
A position *P* is loosely aligned with positions $F_{i}$ and $F_{j}$ if it falls in the green area.

For a given position, *P*, we find the nearest fingerprint positions $F_{i}$ from the set of all fingerprint locations, $F_{i}\in F$. If an arbitrary point in a closed area (P∈A) is *loosely aligned* with two fingerprint locations on which some histograms are defined, we can compute an affine combination of histograms on this point *P* using the two fingerprints. A point *P* is aligned loosely with two other points $\left(F_{i},F_{j}\right)$ if

$$\begin{array}{c} d^{⊥}\left(\langle F_{i},F_{j}\rangle ,P\right)\leq \rho \end{array}$$

where $d^{⊥}$ defines the orthogonal distance of a point to a line, $\langle F_{i},F_{j}\rangle$ is the set of affine combinations or simply the line passing through the points $F_{i}$ and $F_{j}$, and ρ is the *looseness parameter* (See Figure 9).

Finally, if there exist predefined histograms at positions $F_{i}$ and $F_{j}$ and if *P* is aligned loosely with these positions with respect to a previously defined ρ, we can apply an affine Wasserstein combination of histograms between $h^{F_{i}}$ and $h^{F_{j}}$ at *P*.

##### Two-Position Affine Wasserstein Combination (*AWC-B*)

As an attenuation model of wireless signals does not necessarily behave linearly, we propose multiple mappings, $f_{m}$, that map the distance between map positions to the Wasserstein similarity parameter, α. These mappings are linear, quadratic, cubic, logarithmic, and exponential mappings, $\alpha =f_{m}(∥F_{i},P∥)/f_{m}(∥F_{i},F_{j}∥)$. This mapping is another parameter to be decided upon.

With $T_{F_{i},F_{j}}$ being the Wasserstein mapping from the histogram at $F_{i}$ to the histogram at $F_{j}$, the affine combination of histograms between $h^{F_{i}}$ and $h^{F_{j}}$ at *P* is computed. An estimator for the probability density at the position *P* would be:

(12) $$\begin{array}{c} p\left(D_{b}|P,\Theta \right)={\tilde{h}}_{\mathrm{AWC}-B}^{P}\left(\alpha ,\beta \right) \end{array}$$

The original Wasserstein transport problem is defined to be an interpolation [33], where α is restricted to be in [0,1], however without this limitation in α, the interpolation generalizes to an affine combination, so that the position *P* does not necessarily have to reside in the convex hull of the fingerprints. The method is expected to give a likelihood density estimation out of this hull, as the candidate position *P* may exist out of the hull. The definition does not depend on the density of the fingerprints, but the accuracy would depend on the scatter of the fingerprints.

##### Multiple-Position Combination (*AWC-E*)

The Wasserstein combination method for a single position can be employed using only two fingerprints, but the method is prone to losing any other surrounding information. In order to take advantage of the whole map and be able to compare this method with other estimation algorithms that use more than two fingerprints, we develop an extension on the affine Wasserstein combination method.

According to this extension, for the position of interest, *P*, we select all of the possible fingerprint pairs that can generate a combination according to the selection model. For each pair we perform the pairwise combination and obtain a histogram estimation on *P* with each pair. The final combination, ${\tilde{h}}_{\mathrm{AWC}-E}$, is found by taking the affine combination of the available two-position affine Wasserstein combinations, ${\tilde{h}}_{\mathrm{AWC}-B}$, and averaging them with the weights, $\lambda_{i}$, inverse exponentially proportional to the pairwise average distances to *P*. We will denote the final histogram with ${\tilde{h}}_{\mathrm{AWC}-E}$:

(13) $$\begin{array}{c} p\left(D_{b}|P,\Theta \right)={\tilde{h}}_{\mathrm{AWC}-E}^{P}=\sum_{i}\exp\left(-\lambda_{i}\right){\tilde{h}}_{\mathrm{AWC}-B}^{P} \end{array}$$

### 2.4. Inference with the SMC Filter

Having a transition density between positions and an estimate for the observation density to evaluate the measurements with respect to the positions, we have all the ingredients to build up a sequential Monte Carlo method (particle filter) [42,43] to track an agent that reads data from the surrounding beacons. The algorithm of the SMC filter is given in Algorithm 2.

In our setting, we use the generative model defined in (1) with the graphical model in Figure 3. For the transition density, $p(P_{t}|P_{t-1}^{\left(i\right)})$, we employ the diffusion motion model (2), assuming that no rotation or translation information is supplied. For the observation density, $p(D_{t}|{\tilde{P}}_{t}^{\left(i\right)})$, we have five different estimators, listed in Section 2.3 as (4), (5), (8), (12) and (13).

**Algorithm 2** SMC Filter for BLE Localization
1:InstantiateFor i∈[1..N], sample $P_{0}^{\left(i\right)}∼p\left(P_{0}\right)$2:Importance SamplingUpdate time index t←t+1For i∈[1..N], sample ${\tilde{P}}_{t}^{\left(i\right)}∼p\left(P_{t}|P_{t-1}^{\left(i\right)}\right)$For i∈[1..N], evaluate to obtain the importance weights $w_{t}^{\left(i\right)}=p\left(D_{t}|{\tilde{P}}_{t}^{\left(i\right)}\right)$Normalize the importance weights ${\tilde{w}}_{t}^{\left(i\right)}=\frac{w_{t}^{\left(i\right)}}{\sum_{i=0}^{N}w_{t}^{\left(i\right)}}$3:SelectResample *N* particles as $P_{t}^{\left(i\right)}$ from the particles ${\tilde{P}}_{t}^{\left(i\right)}$ according to the importance weights.4:Recurse with repeating 2.


## 3. Setup

### 3.1. Beacons and Sensors

In our measurement setup, we use multiple stationary beacons that serve as message emitters. These are the commercial Bluetooth low-energy beacons supplied by Boni working at around 1 Hz each. The placement of the beacons are shown in colored circles in Figure 2a.

**Figure 10:**
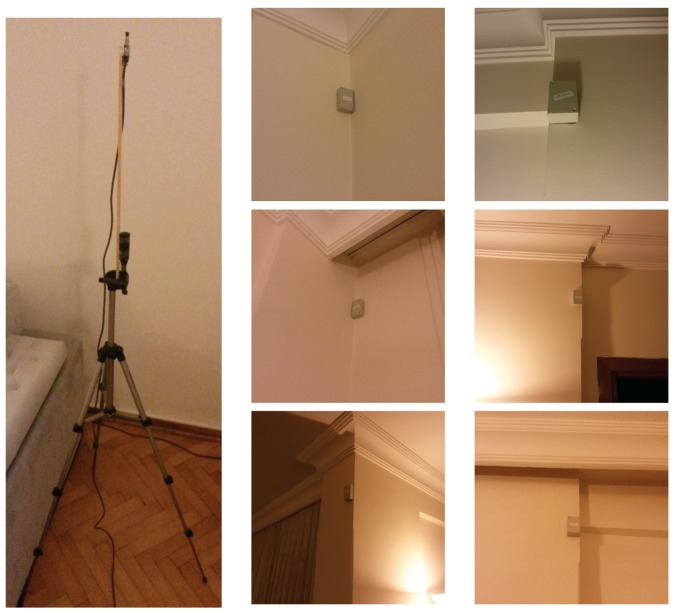
Bluetooth dongle on a tripod and beacons mounted on the walls.

A USB Bluetooth dongle receives the BLE messages and reads RSSI values that are used for both fingerprinting and tracking purposes. The Bluetooth dongle is attached to the top of a tripod (1.5 m in height) to elevate the sensor in order to increase the line-of-sight with the beacons (see Figure 10).

### 3.2. Test Area and Fingerprints

We use a living room of a flat actively used by a married couple to collect data. A plan of the room is given in Figure 2a. The area has a length of 6.66 m and a width of 5.36 m. To gather data, we log the incoming BLE RSSI data with respect to the beacon MAC address on different points in the test area.

On the selected locations of the area, we set up the Bluetooth dongle to dump the incoming raw BLE RSSI data for 24 h, corresponding to about 85 K data points for each beacon, in order to obtain a data spectrum as redundant and as time invariant as possible. Histograms on each position for each dongle are then generated using these data. In our work, a total of 50 days of BLE data are collected with one location per day, making 50 different positions on the map (Figure 2a). Histograms are normalized to represent a density of the RSSI values on a point on the map. If no data belonging to a beacon can be captured on a certain point, then its corresponding distribution is set to constant zero (see Figure 2b). The normalized version of the histograms are called the *fingerprints* of the specific indoor area, which represent a summary of the map in the means of BLE data, or a probabilistic radio map.

This work aims to encode the location (not distance) information with the fingerprints. Hence, a previous scene analysis is required. Should a new beacon be added into the system, its fingerprint information on specific locations should also be added. In our setup, we naturally receive data from other beacons, or other Bluetooth based transmissions. We filter them out as we do not assume to have any previously known “location vs. rssi”-based information related to those beacons.

Because measurements are conducted in an actual living room, where interferences due to Wi-Fi signal from multiple sources, other Bluetooth-based signals, multiple living beings, and metallic masses are highly probable.

### 3.3. Model Parameters

Emission density estimators have their own model parameters (represented previously as Θ). Before employing the tracking algorithms with different estimators, we search for their optimal values by trial-and-error using the leave-one-out cross-validation (LOOCV) strategy [44,45] using the full set of fingerprints (50 measurement locations) and search for the parameter configurations that minimize the error between the estimated histogram and the original fingerprint. We report these parameters as a list:

Nearest fingerprint (NF):
  - No parameters
k-Nearest fingerprints (kNF):
  - Number of nearest fingerprints: k=8
Artificial neural networks with one layer (ANN):
  - Number of hidden layer nodes: $N_{h}=40$
  - Hidden layer activation function: tanh
  - Output layer activation function: sigmoid
Affine Wasserstein combination with the best two positions (AWC-B):
  - Distance to decibel mapping function: linear
  - Evolution type parameter: β=0.36
  - Looseness parameter: ρ=0.63
Affine Wasserstein combination with multiple positions (AWC-E):
  - Distance to decibel mapping function: linear
  - Evolution type parameter: β=0.38
  - Looseness parameter: ρ=0.53

We also search for the optimal variances (*R*) of the predictive distribution for the diffusion model (Section 2.2) that maximizes the localization performance. For this purpose, we take tests of the whole tracking system with different observation density estimators using different variances for the predictive distribution. With an exhaustive search we find and set the optimal variance parameter as R=0.38.

### 3.4. Fingerprint Sets

**Figure 11:**
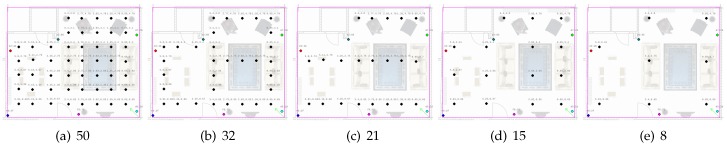
Distribution of fingerprint locations with different sizes. Black dots: measurement locations, colored dots: beacon locations.

We test the observation density estimators on different subsets of fingerprint locations. Beginning with the whole set of 50 fingerprint locations, we run simulations with the estimation algorithms using the sets of 32, 21, 15 and 8 fingerprint locations. The configurations are shown in Figure 11.

As the complexity of the algorithms are also similar, we do not mention their complexities. Only ANN takes too long to train, while it passes an actual training procedure compared to the others. Moreover, we use a fine grid structured probabilistic radio map, on which histograms for every beacon every grid center are previously computed, so that for the actual runs the likelihood estimation of the particles is merely a lookup table operation with the trade-off of high memory usage.

### 3.5. Test Trajectories and Observations

**Figure 12:**
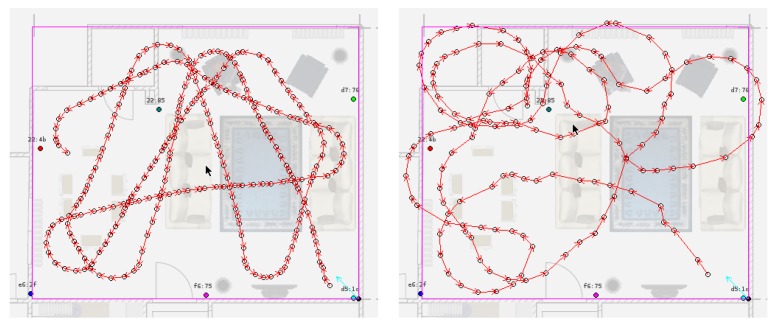
Sampled trajectories with different parameters.

For testing purposes, we sampled synthetic trajectories that imitate the movement of a person that navigates at 0.5 m/s with low tendency to rotate and receives beacon data similar to the BLE RSSI data generated in the setup area (see Figure 12). The details of the trajectory generation are given in Appendix A.

Observational data are sampled afterwards on the trajectory points. The details of the data generation is given in Appendix B.

### 3.6. Summary of Experiments

**Figure 13:**
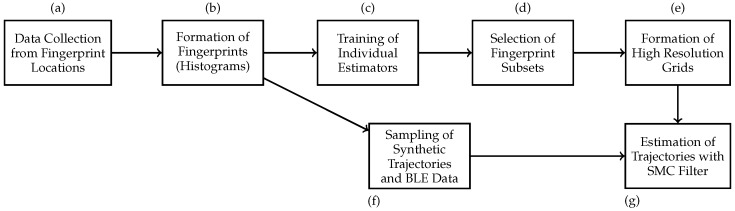
Block diagram of the experimental setup.

We supply a brief summary of the experiments for the readers (see Figure 13). As per the figure, we first collect long hours of BLE RSSI data from densely selected points in the area (a). We then convert these data into fingerprints (normalized histograms) that can be queried for the likelihood of a RSSI value given 2D point and beacon ID (b). With data from multiple fingerprint locations in hand, we search for the model parameters of the individual location estimators like *k* in kNF, $N_{h}$ in ANN or β and ρ in AWC-based estimators (c). We then select a subset of fingerprint locations (d) and we then run the estimators with these parameters on a fine grid with points of 0.1 m apart, in order to construct a grid of observation densities for different beacons, or namely an estimate of the probabilistic radio map of the area (e).

In a different track, (f) we sample a trajectory of a virtual agent that navigates in the test area and generate RSSI data from the previously collected dense fingerprint locations. Finally, using the estimate of the probabilistic radio map as the observation density, sampled RSSI data on the trajectory points as the observations and the motion model as the prediction density, (g) we employ the SMC filter to estimate the position estimations for the observations and measure the tracking performance of the specific method.

## 4. Simulations and Results

**Figure 14:**
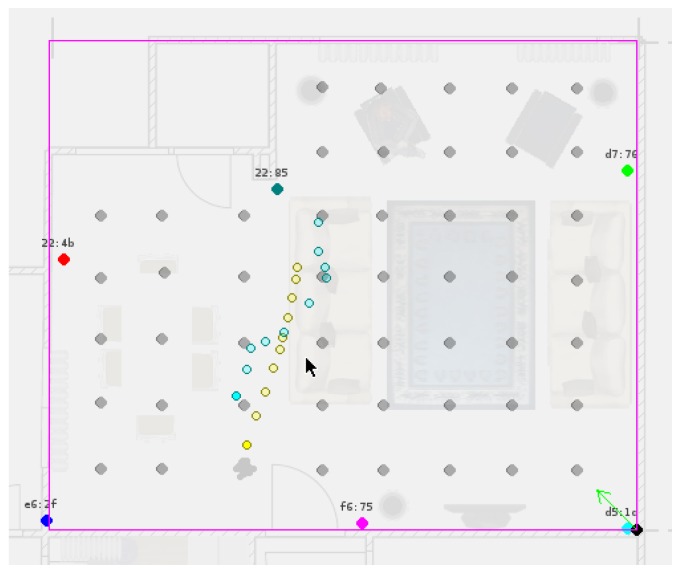
Running sequential Monte Carlo (SMC) filter: The yellow trajectory shows the computer-generated positions, while the cyan trajectory shows the estimation.

We defined five different estimator methods to estimate the observation densities: NF, kNF, ANN, AWC-B, and AWC-E. The estimations are performed using 50, 32, 21, 15, and 8 measurement locations. The estimators estimate the observation densities for particle evaluation in the update step of the SMC algorithm. For the prediction step we employ the motion model defined in Section 2.2, and the recursive loop of the SMC algorithm is closed. The simulations are designed with Python-3.4 and are run with a particle size of 10 K on an Intel Xeon 2630. The particle evaluation step is parallelized by 32 processes. Each iteration takes about 0.8 s. A snapshot of the running SMC algorithm is given in Figure 14.

**Figure 15:**
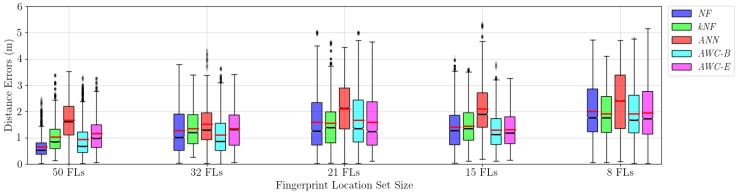
Trajectory estimation performances of different observation models with different sizes of fingerprint sets with black lines denoting medians and red lines for means. *NF*: nearest fingerprint; *kNF*: k-nearest fingerprint; *ANN*: artificial neural network; *AWC-B*: affine Wasserstein combination with the best two positions; *AWC-E*: affine Wasserstein combination with multiple positions.

The SMC filter was run for the same trajectory of 232 points, with 32 of them discarded as the burn-in period, leaving us with 200 points of error for each run. We log the distances of the predicted positions to the original positions as errors. Each combination (estimator and fingerprint set size) is repeated 30 times, which gives 6000 position estimations or error measurements per combination. The box plot of the statistics is given in Figure 15. We report both mean and median errors.

It can be seen from the results that high-resolution fingerprint information with 50 fingerprint locations (FL) defines the map the best for the tracking purposes with any of the applied methods. Without employing any complicated method, that is, using only NF in estimating the observation density, gives the best result: an error rate of 0.66 m, with the lowest variance. This is an expected result but with an impractical setting. With more than 50 fingerprint locations, this error rate will surely get lower, but making the scene analysis much more of a burden at the same time. Collecting data from many locations is obviously not practical. A reduced number of fingerprints would facilitate the installation and calibration procedure in BLE positioning and tracking infrastructure, so that the results for such scenarios are substantial and realistic for us.

As the configurations lose a number of fingerprint locations, we see that NF is unable to keep its success with the highest resolution, and Wasserstein interpolation-based techniques stand out. Even though the error rates are over 1 m, AWC-B looks especially promising with an error rate of 1.29 m with 15 fingerprint locations. The same algorithm gives an error rate of 1.9 m with only 8 fingerprint locations.

The neural network approach (ANN) is both inconvenient as we do not have many samples for training and impractical as the training iterations take too long. Moreover, the results show that ANN yields consistently higher error rates compared to the other methods. These results are expected whilst the neural networks require many samples beforehand, and we also try to reduce the required fingerprint size, which makes ANN naturally inappropriate for our purposes.

Table 1 summarizes the mean localization errors for different fingerprint confugurations with respect to the applied estimator algorithms. Concentrating on the results with lower number of fingerprints (15 and 8), we see that Wasserstein-based methods race head to head with the kNF method. We also run two *t*-tests for significance analysis on the last two configurations. In the significance test tables we report the *p*-values for the one-sided hypothesis if the value on the row is less than the value on the column. According to the *p*-values, for 15 fingerprint locations (see Table 2), AWC-B has the best localization performance, but for the configuration of 8 fingerprint locations (see Table 3), there is no best estimator, because AWC-B and kNF cannot outperform each other.

**Figure 16:**
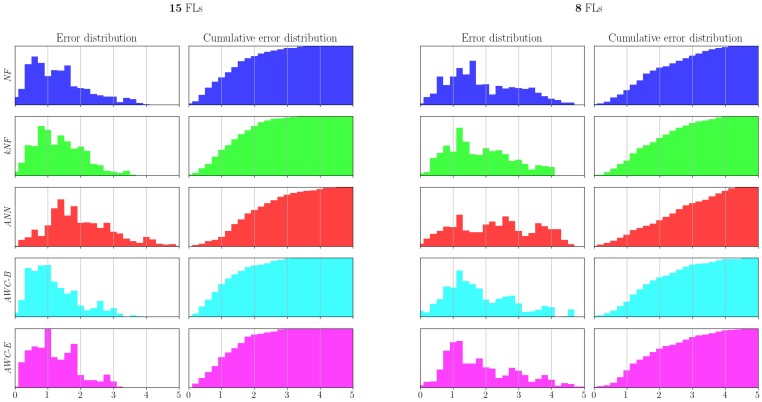
Localization error distributions and corresponding cumulative error distributions for two fingerprint location configurations: 15 and 8 locations.

Even though the box plots in Figure 15 give a hint about error distributions, we also supply the explicit distributions and corresponding cumulative error distributions. In Figure 16, we encode the methods with the same color codes with Figure 15.

The figures clearly show that the error distributions are not normally distributed, which is probably due to sudden incorrect far position estimations in the SMC filter. We believe that such results are better shown with box plots and medians, as the errors have skewed distributions, where means would be misleading. We also report the error medians in Table 4.

For the significance test, because the error results are not necessarily normally distributed, we apply one-sided Wilcoxon signed-rank tests on the error rates of the configuration pairs. We report the two low sized fingerprint sets’ values with the confidence value of α<0.05.

Table 5 shows that for the configuration of 15 fingerprint locations, the proposed methods AWC-B and AWC-E, perform significantly better than any other methods we compared. For a smaller configuration of eight locations, we see almost the same results. Table 6 shows that the proposed methods perform significantly better than the applied techniques, except against kNF, in which we can say that our methods perform slightly better than kNF. This leads to the claim that, for the small-sized fingerprint sets (8 and 15), Wasserstein-based interpolation techniques reduce the errors significantly, (except for only one case) compared to the applied techniques so far. Moreover, as the complexity of the methods is similar, Wasserstein interpolation techniques are preferable for small-sized fingerprint sets. Amongst the proposed methods, AWC-B is seen to perform slightly better than its counterpart AWC-E.

Thus, we conclude that that two methods AWC-B and AWC-E are the two candidates to be used in observation density estimation in the SMC filter for tracking purposes with BLE beacon information for lower number of fingerprints if the positions of these fingerprints are scattered evenly in order to perform better histogram interpolations.

## 5. Conclusions

In this work, we have developed a method to render indoor localization and tracking practical using only BLE sensors. Our model-based approach relies critically on the accurate estimation of a probabilistic radio map—a distribution of RSSI values for every position inside the region of interest—from a few RSSI fingerprint measurements, obtained only at a few locations. The estimated radio map is subsequently used as the observation model of a dynamical system where we do target tracking by a sequential Monte Carlo algorithm.

Not surprisingly, there is a direct relationship between the accuracy of the radiomap and accuracy of localization and tracking. We show first that when RSSI fingerprints are collected on a very dense grid, a radio map can be accurately estimated and a satisfactory tracking performance can be obtained despite the high variability of the actual measurements. However, dense fingerprint sampling is often not practical or at least not desired in real applications as this requires careful measurement and a long data collection process. In this paper, we develop methods to obtain accurate radio maps from far fewer and sparsely sampled fingerprints and show empirically that tracking accuracy is still acceptable.

We cast the radio map estimation as a fingerprint interpolation method, that we cast as a regression problem. We have proposed two variations of the Wasserstein interpolation method, which is also originally derived from the Wasserstein distance computation method. The first one uses two points as the pivot points to find an affine combination on an unknown coordinate aligned with the pivot points. The second one fuses multiple affine combinations to estimate a histogram on an unknown coordinate. The results of these Wasserstein variations are compared with the results of the well known regression methods, namely the nearest neighbor approach, the affine combination and the neural networks

We can generalize from the results that we can estimate the unknown radio information on the arbitrary positions by an interpolation using the radio strength densities on previously measured positions. Moreover, Wasserstein combination variations are good candidates for histogram estimation purposes, as the error rates of these metods increase consistently while the number of fingerprints are reduced, but they perform better compared to the other applied techniques.

An expected result is that with such small data, neural networks are unable to learn the nonlinear histogram on a planar surface without overlearning. The domain can benefit from the autoregressive models to train the histograms against the positions.

In fingerprinting techniques, initial scene analysis is a must, but it is not practical to collect data from high number of locations. For future work, an immediate study could to perform an analysis on the appropriate fingerprint locations. Having the practicality in mind, the researcher can also find the number of fingerprint locations with respect to the area size and the required fineness. Additionally, if a method can give confidence for a position in the area, it may urge the researcher to gather data from locations with low confidence values. A proper Gaussian process regression for multiple outputs would be a good future study using this data, as the algorithm by nature provides the confidence values.

## Figures and Tables

**Table 1:** Means of trajectory estimation performances.

Means	50 FLs	32 FLs	21 FLs	15 FLs	8 FLs
NF	0.663	1.266	1.602	1.414	2.012
kNF	1.033	1.359	1.561	1.445	1.908
ANN	1.661	1.515	2.098	2.094	2.395
AWC-B	0.932	1.098	1.662	1.294	1.910
AWC-E	1.157	1.355	1.588	1.314	1.956

**Table 2:** *p*-values of the pairwise one-sided *t*-tests of the results for the set of 15 FLs.

<	NF	kNF	ANN	AWC-B	AWC-E
NF	-	0.0000	0.0000	1.0000	1.0000
kNF	1.0000	-	0.0000	1.0000	1.0000
ANN	1.0000	1.0000	-	1.0000	1.0000
AWC-B	0.0000	0.0000	0.0000	-	0.0000
AWC-E	0.0000	0.0000	0.0000	1.0000	-

**Table 3:** *p*-values of the pairwise one-sided *t*-tests of the results for the set of 8 FLs.

<	NF	kNF	ANN	AWC-B	AWC-E
NF	-	1.0000	0.0000	1.0000	1.0000
kNF	0.0000	-	0.0000	1.0000	0.0000
ANN	1.0000	1.0000	-	1.0000	1.0000
AWC-B	0.0000	1.0000	0.0000	-	0.0000
AWC-E	0.0000	1.0000	0.0000	1.0000	-

**Table 4:** Medians of trajectory estimation performances.

Medians	50 FLs	32 FLs	21 FLs	15 FLs	8 FLs
NF	0.519	1.008	1.259	1.267	1.754
kNF	0.847	1.194	1.385	1.342	1.753
ANN	1.607	1.289	2.127	1.889	2.403
AWC-B	0.676	0.859	1.349	1.118	1.672
AWC-E	0.984	1.309	1.232	1.174	1.724

**Table 5:** *p*-values of the pairwise one-sided Wilcoxon tests of the results for the set of 15 FLs.

<	NF	kNF	ANN	AWC-B	AWC-E
NF	-	0.0000	0.0000	1.0000	0.9998
kNF	1.0000	-	0.0000	1.0000	1.0000
ANN	1.0000	1.0000	-	1.0000	1.0000
AWC-B	0.0000	0.0000	0.0000	-	0.0001
AWC-E	0.0002	0.0000	0.0000	0.9999	-

**Table 6:** *p*-Values of the pairwise one-sided Wilcoxon tests of the results for the set of 8 FLs.

<	NF	kNF	ANN	AWC-B	AWC-E
NF	-	1.0000	0.0000	1.0000	1.0000
kNF	0.0000	-	0.0000	0.9014	0.5156
ANN	1.0000	1.0000	-	1.0000	1.0000
AWC-B	0.0000	0.0986	0.0000	-	0.1205
AWC-E	0.0000	0.4844	0.0000	0.8795	-

## Appendix A. Trajectory Sampling

The generative model from which we sample the trajectory points follows:

(A1) $$\begin{array}{c} \begin{array}{cc} \tau_{0} & =0\\ x_{0},y_{0} & ∼U(A)\\ \theta_{0} & ∼U([0,2\pi ])\\ {\tilde{\delta }}_{t}^{\tau } & ∼G(a_{\tau },b_{\tau })\\ {\tilde{\delta }}_{t}^{l} & ∼G(a_{l},b_{l})\\ {\tilde{\delta }}_{t}^{\theta } & ∼N(0,s)\\ \tau_{t} & =\tau_{t-1}+{\tilde{\delta }}_{t}^{\tau }\\ x_{t} & =x_{t-1}+{\tilde{\delta }}_{t}^{l}\cos\left(\theta_{t-1}+{\tilde{\delta }}_{t}^{\theta }\right)\\ y_{t} & =y_{t-1}+{\tilde{\delta }}_{t}^{l}\sin\left(\theta_{t-1}+{\tilde{\delta }}_{t}^{\theta }\right)\\ \theta_{t} & =\theta_{t-1}+{\tilde{\delta }}_{t}^{\theta } \end{array} \end{array}$$

We sample an initial location in A and an initial orientation between 0 and 2π, and set the initial time stamp to 0. At each step, a time interval, ${\tilde{\delta }}_{t}^{\tau }$, and a distance value, ${\tilde{\delta }}_{t}^{l}$, are sampled from gamma distributions, and a rotation value, ${\tilde{\delta }}_{t}^{\theta }$, is sampled from a normal distribution. The parameters of the distributions, $a_{\tau },b_{\tau },a_{l},b_{l},s$ are experimentally set to generate values similar with the real data. To prevent our virtual robot from leaving the area, sampled rotation values are deliberately manipulated by an additional value of $\frac{\pi }{8}$ according to the current orientation. This helps the robot return smoothly into the area when it approaches the borders. Sample trajectories can be seen in Figure 12.

## Appendix B. Data Sampling

The generative model from which we sample the RSSI data on the trajectory points follows:

(A2) $$\begin{array}{c} \begin{array}{cc} b_{t} & ∼M(b,P_{t})\\ i^{*} & =\arg\min_{i}d\left(P_{t},F_{i}\right)\\ D_{t} & ∼M(h_{b_{t}}^{F_{i^{*}}}) \end{array} \end{array}$$

We first sample a beacon label from the set of all available beacons b, according to a probability distribution $P_{t}$, which reshapes at each time step inversely proportional to the use counts of the beacons, in order to let the beacons be sampled uniformly in small time windows. We then find the closest fingerprint index ($i^{*}$), and sample an RSSI value, $D_{t}$, using the corresponding beacon histogram.
